# Novel Zirconia Surface Treatments for Enhanced Osseointegration: Laboratory Characterization

**DOI:** 10.1155/2014/203940

**Published:** 2014-09-29

**Authors:** Ola H. Ewais, Fayza Al Abbassy, Mona M. Ghoneim, Moustafa N. Aboushelib

**Affiliations:** ^1^Dental Biomaterials Department, Faculty of Dentistry, Alexandria University, Alexandria, Egypt; ^2^Biomaterials Department, Faculty of Dentistry, Alexandria University, Alexandria, Egypt; ^3^Conservative Dentistry Department, Faculty of Dentistry, Alexandria University, Alexandria, Egypt

## Abstract

*Purpose*. The aim of this study was to evaluate three novel surface treatments intended to improve osseointegration of zirconia implants: selective infiltration etching treatment (SIE), fusion sputtering (FS), and low pressure particle abrasion (LPPA). The effects of surface treatments on roughness, topography, hardness, and porosity of implants were also assessed. *Materials and Methods*. 45 zirconia discs (19 mm in diameter × 3 mm in thickness) received 3 different surface treatments: selective infiltration etching, low pressure particle abrasion with 30 *µ*m alumina, and fusion sputtering while nontreated surface served as control. Surface roughness was evaluated quantitatively using profilometery, porosity was evaluated using mercury prosimetry, and Vickers microhardness was used to assess surface hardness. Surface topography was analyzed using scanning and atomic force microscopy (*α* = 0.05). *Results*. There were significant differences between all groups regarding surface roughness (*F* = 1678, *P* < 0.001), porosity (*F* = 3278, *P* < 0.001), and hardness (*F* = 1106.158, *P* < 0.001). Scanning and atomic force microscopy revealed a nanoporous surface characteristic of SIE, and FS resulted in the creation of surface microbeads, while LPPA resulted in limited abrasion of the surface. *Conclusion*. Within the limitations of the study, changes in surface characteristics and topography of zirconia implants have been observed after different surface treatment approaches. Thus possibilities for enhanced osseointegration could be additionally offered.

## 1. Introduction

Developments in routine dental practice, including prosthodontic treatments, are often driven by the introduction of new dental materials and processing technologies [1].

Zircon has been known as a gem from ancient times. The name of the metal, zirconium, comes from the Arabic* Zargon *(golden in colour) which in turn comes from the two Persian words* Zar *(Gold) and* Gun *(Colour). Zirconia, the metal dioxide (ZrO_2_), was identified in 1789 by the German chemist Martin Heinrich [2].

The crystalline form of zirconia can be organized in three different crystal structures; at room temperature, zirconia adopts a monoclinic (M) structure and transforms into the tetragonal phase (T) at 1170°C, followed by a cubic phase (C) at 2370°C. During cooling, a phase transformation from T to M takes place and is associated with a volume expansion of 3-4%. The conversion of the tetragonal to the monoclinic phase leads to the formation of surface cracks that might result in surface degradation or bulk failure of bulk material [3].

In order to obtain mechanical stability at room temperature, addition of different metallic oxides, such as MgO, CaO, or Y_2_O_3_, stabilizes the tetragonal phase at room temperature resulting in fully or partially stabilized zirconia (PSZ) [4]. The high resistance of PSZ against crack propagation is based on a phase transformation from the tetragonal to the monoclinic phase and its associated expansion, a mechanism known as transformation toughening [3, 5].

In dental implantology, the development of yttria-stabilized tetragonal zirconia polycrystal ceramics (Y-TPZ) gained a great interest as a prosthetic and implant material. Y-TPZ has a higher fracture resistance and flexural strength than the previously available aluminium oxide ceramics, making it less sensitive to stress concentrations. It also exhibits a moderate Young's modulus (200 GPa) in comparison to aluminium oxide, indicating a higher elastic deformation capability [6].

Y-TZP has been used extensively in orthopedic surgery as a material for ball heads in total hip replacement since its introduction in the 1980s [7]. In dentistry, Y-TZP was successfully introduced as a framework material for the construction of all-ceramic fixed partial denture restorations [8]. Today, many types of zirconia materials are available, but yttria-stabilized tetragonal zirconia polycrystals (Y-TZP) are the most widely used [9]. This yttria-stabilized material presents various interesting characteristics such as low porosity, high density, high bending, and compression strength suitable for biomedical applications. Moreover, its bright color offers esthetic benefits, which makes zirconia a very popular material in prosthetic dentistry and dental implantology [10].

To improve surface properties of zirconia implants, two main approaches were used: changing the surface chemistry using bioactive coating with different materials (calcium phosphates, bisphosphonate, collagen, etc.) and optimizing surface architecture and microroughness using different techniques [11]. Airborne particle abrasion known as sandblasting technique is the most commonly used technique to increase surface roughness of zirconia. However, it was associated with noticeable surface damage in the form of scratches and grooves resulting in marked reduction in mechanical properties [12]. To solve this issue, low pressure particle abrasion (LPPA) modified the application parameters to include low air pressure and reduced blasting time, using small particle sizes in order to minimize surface damage [12].

Selective infiltration etching technique (SIE) is a recent method used to create surface roughness of zirconia on a nanoscale (less than 0.05 *μ*m) using principles of heat-induced maturation and grain boundary diffusion to transform the relatively smooth nonretentive surface of zirconia into a highly porous and retentive surface [13].

Instead of abrading the surface of zirconia, fusion sputtering is a simple technique used to increase surface roughness of zirconia and to change surface architecture by the creation of round surface beads fused with surface of the material. Green body zirconia, unsintered structure, is sprayed with a solution rich in round zirconia particles. Upon sintering, the particles become fused with the sprayed structure. Only short-term results are available about the performance of fusion sputtered zirconia implants [14].

Implants with a rough surface yielded both greater shear strengths and superior bone apposition compared to implants with smooth surfaces which exhibited various degrees of fibrous tissue encapsulation [15, 16]. Microscale roughness and micron-sized topography have been shown to have an essential role in the induction of bone cell adhesion and subsequent changes in cellular function [17].

Unfortunately, there are not enough works of research done about the expected biological response of bone tissue to zirconia implants with different surface treatments. The aim of this study was to investigate and characterize three novel surface treatments of zirconia implants: selective infiltration etching, low pressure particle abrasion, and fusion sputtering to evaluate osseointegration in a separate study.

## 2. Materials and Methods

Three novel surface treatments were performed on 45 zirconia discs that were divided into 3 groups, 15 each, according to the surface treatment performed. Surface properties and topography were evaluated using different laboratory tests. The same surface treatments, in a separate study, were performed on zirconia implants in order to assess osseointegration in the femur of a rabbit model.

### 2.1. Fabrication of Zirconia Specimens

A special brass mold (19 mm in diameter × 3 mm in thickness) was used for isostatically pressing zirconia powder (3 mol Y-TZP, E grade biomedical zirconia, Tosoh Inc., Japan). 60 discs were sintered in special electrical furnace (Cercon heat, DeguDent GmbH, Hanau, Germany) with a sintering program at a maximum temperature of 1350°C for 4 hours. The discs, thereafter, received three different surface treatments. The discs received one of the following surface treatments while as-sintered discs served as control (*n* = 15).

### 2.2. Selective Infiltration Etching (SIE) [13]

One surface of the discs was coated with a thin layer of an infiltration agent composed of a low temperature melting glass with different additives that control its viscosity and thermal expansion coefficient (10.1 × 10^−6^/°C). The discs were then heated in open air at 850°C for 2 min using a computer programmed electrical induction furnace and then cooled to room temperature by opening the door of the furnace. Traces of the infiltration agent were completely dissolved in a 5% hydrofluoric acid solution in an ultrasonic bath for 15 minutes, followed by washing under demineralized water for 15 minutes.

### 2.3. Low Pressure Particle Abrasion (LPPA) [12]

The discs received airborne particle abrasion with 30 *μ*m aluminum oxide particles at 0.15 MPa pressure at a distance of 3 cm in a special sandblasting machine (Percision Dental Laboratory Sandblaster P-G 400; Winterbach, Baden-Wurttemberg, Germany). To control the procedure, a blasting rate of 25 s/cm^2^ was used. Specimens were ultrasonically cleaned in distilled water for 15 minutes.

### 2.4. Fusion Sputtering Technique (FS) [14]

Five grams of unsintered (Y-TZP) powder (Tosoh zirconia, E grade, Tokyo, Japan) was placed in a plastic capsule, with 1 mm zirconia balls. The sealed capsule was placed in an electric mixer for 15 minutes to allow fine grinding of the zirconia powder. Only 12–18 *μ*m particles were selected by the use of fine stainless steel meshes. 30 grams of the selected particles was added to a glass jar filled with 150 mL of 70% ethyl alcohol and 30 gm of polyvinyl glycol. Thereafter, the mixture was placed on an ultrasonic shaker to allow homogenous distribution of the particles. The suspension was transferred to a spraying container with air pressure set at 0.3 MPa (Badger 155-19 Anthem 155 Airbrush Set w/Modelflex Paints, USA). One surface of unsintered zirconia discs was sprayed for 5 seconds. The surface sputtered discs were then stored at 60°C for 2 hours to allow proper drying of surface before sintering.

### 2.5. Profilometer Analysis

The surface roughness of the discs was measured using a contact profilometer (Taylor Hobson Precision Form Talysurf 60, Leicester, UK). The specimens were mounted on an XY cross-table of the profilometer, and three tracings 5 mm in length and separated by a total distance of 140 *μ*m were evaluated. The following roughness parameters were evaluated:

- amplitude parameters (bi-dimensional surface structure),
- *R*_*a*_ value which is the arithmetical mean of the absolute values of the surface departures from the mean plane within the sampling area (in *μ*m),
- *R*_*q*_ value which is the root mean square value of the surface departures within the sampling area (in *μ*m). This parameter is more sensitive to extreme values than the *R*_*a*_ parameter due to the squaring operation,
- *R*_*z*_ value which is the average value of the absolute heights of the five highest peaks and the absolute value of the five deepest valleys within the sampling area.

### 2.6. Porosity and Density Measurements

Specimens were tested for porosity percentage using the mercury porosimetry apparatus (QUANTACHROME Instruments 1900, Corporate Drive Boynton Beach, Florida, USA). This was done by immersing the specimens in a mercury bath and then pressure (up to 4000 bar) was applied, thus forcing nonwetting mercury into smaller and smaller pores of the disc surfaces. The pressure required to intrude the mercury into the pores is inversely proportional to the size of the pores. An intrusion volume was recorded at each point and converted to pore size. Bulk and surface density were also calculated.

### 2.7. Vickers Microhardness Test

Vickers microhardness tester (Instron Wolpert HMV-2000, Wolpert Wilson Instruments, USA) used a diamond indenter, in the form of a right pyramid with a square base and an angle of 136 degrees between opposite faces subjected to a load of 10 kg. The full load was applied for 15 seconds. The two diagonals of the indentation after removal of the load were measured using an optical microscope and their average was calculated. The area of the sloping surface of the indentation was calculated. The Vickers hardness was the quotient by dividing the load by the square area of indentation:

(1) $$\begin{array}{c} \text{HV}=\frac{F}{d^{2}}, \end{array}$$

where HV = the Vickers hardness value, *F* = load in kg, and *d* ^2^ = arithmetic mean of the two diagonals,* d1* and* d2,* in mm.

### 2.8. Scanning Electron Microscopy

The surface morphology and topography created by different surface treatments were examined by scanning electron microscopy (SEM) (Jeol, JSM-5300, Tokyo, Japan) performed at various magnifications under an acceleration voltage of 15 keV.

### 2.9. Atomic Force Microscopy

The surface morphology and topography on a nanoscale were examined with atomic force microscope (AFM) (THERMO MICROSCOPES Auto probe AP-0100-contact mode, USA). A surface three-dimensional (3D) imaging was collected in contact mode.

Statistical analysis was performed using one-way analysis of variance (ANOVA) and Bonferroni post-hoc tests (*α* = 0.05) using a computer software (SPSS 15.0, SPSS, Chicago, IL).

## 3. Results

**Figure 1:**
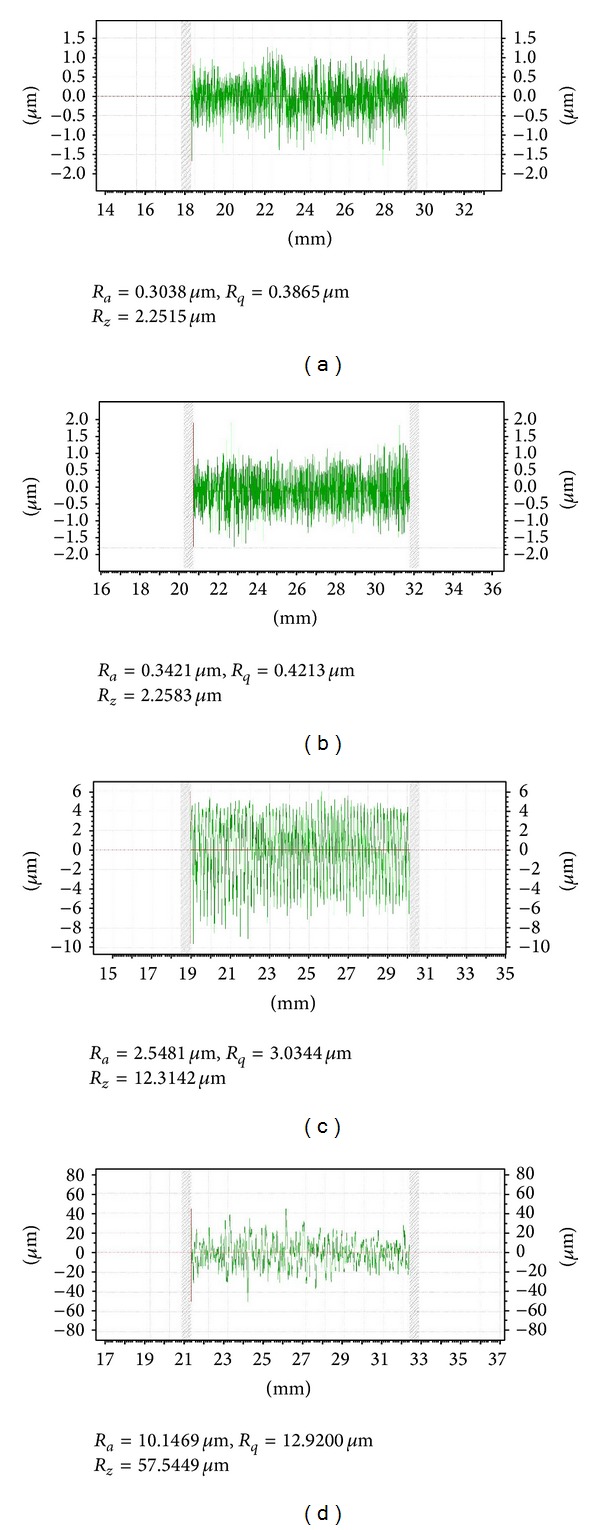
(a) Surface roughness parameters of as-sintered zirconia surface. (b) Surface roughness parameters of selective infiltration etched zirconia surface. (c) Surface roughness parameters of LPPA zirconia surface. (d) Surface roughness parameters of fusion sputtered zirconia surface.

Statistical analysis revealed significant differences in surface roughness parameters between the tested groups (*F* = 1678, *P* < 0.001). FS and LPPA increased the surface roughness compared to as-sintered surface, while SIE failed to increase the surface microroughness. Roughness parameters are summarized in Table 1. Profilometer graphs are depicted in Figure 1.

There were significant differences in pore area (*F* = 131039, *P* > 0.001), pore diameter (*F* = 14, *P* < 0.001), bulk density (*F* = 192422, *P* < 00.01), and porosity percentage (*F* = 3278, *P* < 0.001), as shown in Table 1. There were also significant differences in VH values between the four groups as the lowest hardness value was for as-sintered surface (920 ± 7.1), followed by SIE (1346 ± 11.2), and then LPPA (1608 ± 28.6), and the highest was for FS (2137 ± 68); see Table 1.

**Figure 2:**
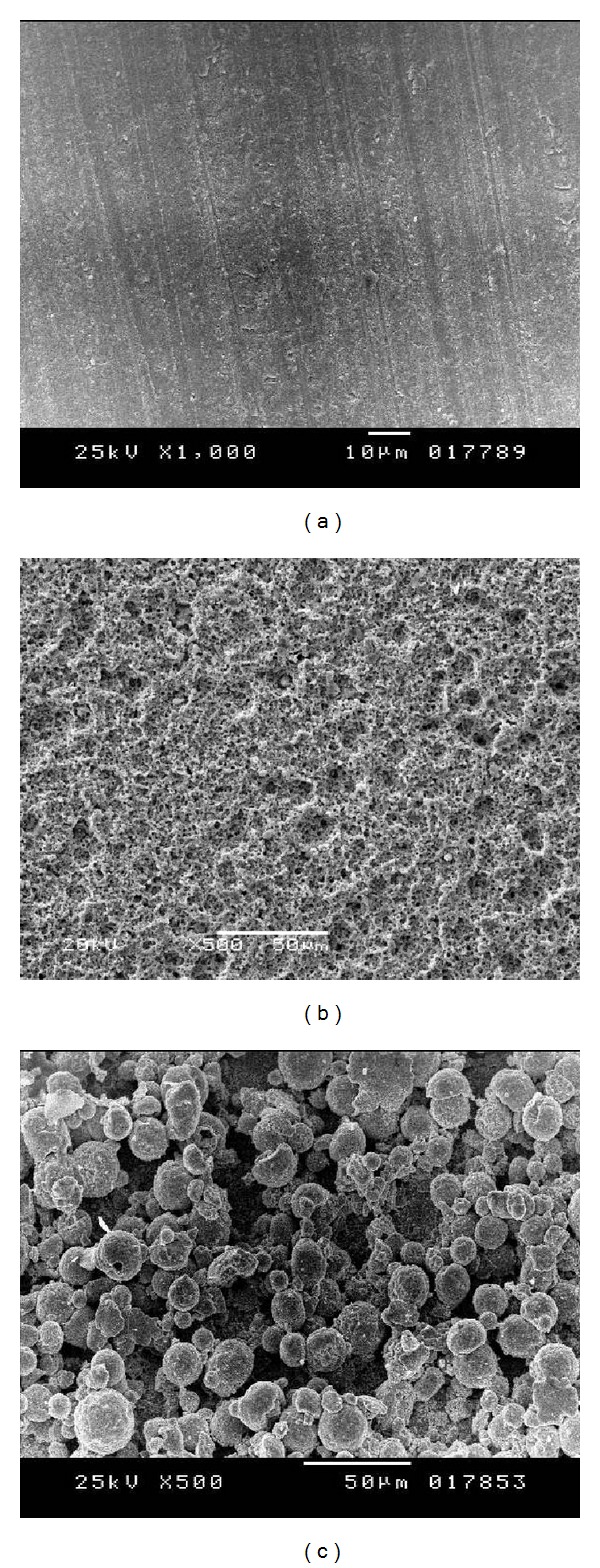
(a) SEM image, 1000x, demonstrating dense nonretentive as-sintered surface zirconia. (b) SEM image, 1000x, of selective infiltration etched zirconia surface with characteristic nanoporous surface. (c) SEM image, 500x, of fusion sputtered zirconia surface with characteristic fused beads on the surface.

SEM examination of as-sintered zirconia surface revealed parallel lines created by fine polishing procedure. Selective infiltration etched specimens had a characteristic nanoporous surface without prominent surface changes in surface roughness; some areas pores, elevations, and depressions were observed. LPPA images indicated the presence of microscratches, grooves, and surface abrasions. The entire surface was roughened but in an irregular pattern. Fusion sputtered specimens had a granular microrough surface covered with rounded irregular zirconia particles fused to the outer surface of the specimens. The surface granules had an average height of 10 *μ*m, which accounts for the increased surface roughness measurements (*R*_*a*_ = 10.23 ± 0.09). Surface granules demonstrated even distribution and an identical morphological pattern along the entire surface, as shown in Figure 2.

**Figure 3:**
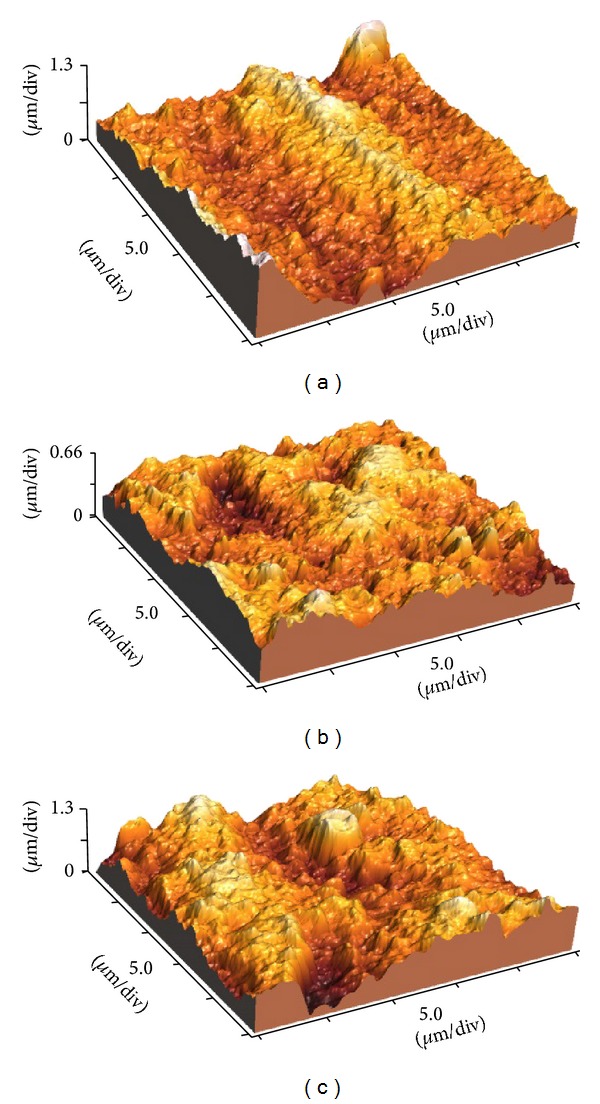
(a) AFM image for as-sintered zirconia surface. (b) AFM image of selective infiltration etched surface. (c) AFM image for fusion sputtered surface.

Three-dimensional (3D) imaging collected by the AFM created an examination field of 25 × 25 *μ*m. The small examination field presented morphological description on a nanoscale, as shown in Figure 3.

## 4. Discussion

Yttria-stabilized tetragonal zirconia polycrystal ceramic is the most commonly used zirconia type by dental manufacturers [18]. The increasing use of all-ceramic restorations has resulted in a heightened demand for improvements in properties and reliability [19]. To improve surface properties of zirconia implants, three novel surface treatments were used. Two resulted in optimizing the microroughness (LPPA and FS) and one optimized nanoroughness (SIE).

Mechanical properties depend not only on the microstructure but also on the size and distribution of structural defects that, in turn, are dependent on the characteristics of the starting powders and on the manufacturing process [2]. Surface texture is believed to play a major role in affecting the strength and clinical survival rate of the restoration [20]. Aggressive surface roughening techniques resulted in the creation of surface defects ending in marked deterioration of the mechanical properties of the restoration [13].

Selective infiltration etching technique created a nanoroughened surface with mean roughness of 0.38 ± 0.04 *μ*m and an increase in the total surface area of the specimen without creation of any structural defects [13, 21]. Numerous studies have treated zirconia with different airborne particle abrasion systems [22, 23]. In this study, another novel treatment was introduced: low pressure particle abrasion technique (LPPA) using 30 *μ*m aluminum oxide particles at 0.15 MPa pressure resulting in microroughened surface with mean roughness of 2.31 ± 0.15 *μ*m; while, in previous studies, sandblasting was carried out using coarser parameters as 110 *μ*m alumina particles applied perpendicularly to the surface and at higher pressure (2-3 bar) [20]. In this study, a microroughened surface was observed due to the low pressure particle abrasion technique (LPPA).

Regarding particle size, Queiroz et al. noticed a large difference between *R*_*a*_ and *R*_*z*_ values in particle abraded specimens due to the presence of deep valleys in the surface for tested specimens. When the 145 *μ*m alumina particles were used, *R*_*z*_ achieved the highest value, suggesting that large particles promoted more punctual damage in a surface than small particles, regardless of the pressure [24].

Frequently, *R*_*a*_ value has been used to express changes in zirconia surface in dental literature. However, the mean roughness (*R*_*a*_) associated with 2D surface images only provides limited information and can lead to an erroneous interpretation of surface roughness. *R*_*a*_ cannot detect differences in the spacing of surface irregularities (peaks and valleys); thus, it cannot provide information regarding their shape [25]. In the current study, *R*_*a*_, *R*_*q*_, and *R*_*z*_ were measured to provide a better insight of surface topography.

New conditioning strategies have been recently proposed to enhance the surface roughness of zirconia in order to promote adhesion of resin cements to zirconia. Serkan et al. [18] applied a low pressure air abrasion (0.05 MPa/0.5 bar) before testing the bonding effectiveness of a conventional luting resin to the treated ceramic putting in mind the reduction of surface damage [26, 27].

Analysis of porosity tests indicated the presence of a direct relationship between porosity and density using correlations between different parameters as pore area, pore diameter, bulk density, and porosity percentages putting in mind that only the pores that are connected to the surface can be measured [28]. On the other hand, He et al. proved that surface roughness decreases with decreasing porosity and that the presence of porosity generally has a negative influence on mechanical properties since pores can cause stress concentration and deterioration of fracture resistance [29]. This explains why porosity created by SIE was comparable to the as-sintered surface.

Surface porosity could be used as a carrier of bioactive materials on the surface of zirconia implants. This coating technique could solve delamination problems observed when coat material is subjected to body fluids. Combination of chemical coat and optimized microrough surface could enhance performance of zirconia implants, the data presented in the second part of this study [30].

The surface microhardness (VHN) in the current study was in the range of 920–2173. In general, there were significant differences in microhardness between all groups as the increase in surface roughness was associated with the increase in surface hardness. This finding was in disagreement with previous studies which reported that an increase in porosity and roughness of material was associated with low surface hardness [2, 31]. Fusion sputtering was associated with an observable increase in surface hardness; this is directly associated with fusion of round surface beads which increase surface capacity to resist the applied load [31].

The conventional sand blasting method with large particles (>100 *μ*m) caused massive sputtering on the Y-TZP surface; thus, air-abrasion as LPPA technique used in current study with small-sized particles (30 *μ*m) should be considered due to the potential reduction in flexural strength and possible material loss with bigger particles, particularly along the margins of restorations [32, 33]. In a different line, Kern et al. applied a low pressure air abrasion (0.05 MPa/0.5 bar) before testing the bonding effectiveness of a conventional composite luting agent to the so-treated ceramic in order to reduce the surface damage. New conditioning strategies have been recently proposed [26].

## 5. Conclusions

Within the limitations of this study, surface topography and architecture of zirconia implants could be optimized using different surface treatments.

## Figures and Tables

**Table 1:** Surface parameters of different test groups.

			Mean	Std. deviation	95% confidence interval for mean		Minimum	Maximum
					Lower bound	Upper bound		
*R*_*a*_	As-sintered		0.2883	0.01722	0.2703	0.3064	0.26	0.31
	SIE		0.3883	0.03710	0.3494	0.4273	0.34	0.44
	LPPA		2.3100	0.14670	2.1561	2.4639	2.12	2.55
	FS		10.2300	0.09274	10.1327	10.3273	10.10	10.35
	Total		3.3042	4.16756	1.5444	5.0640	0.26	10.35
	Model	Fixed effects		0.08915	3.2662	3.3421		
		Random effects			−4.1905	10.7989		

*R*_*q*_	As-sintered		0.3800	0.02449	0.3543	0.4057	0.35	0.42
	SIE		0.4767	0.04633	0.4280	0.5253	0.42	0.54
	LPPA		2.9600	0.04940	2.9082	3.0118	2.89	3.03
	FS		13.0000	0.09274	12.9027	13.0973	12.87	13.12
	Total		4.2042	5.29425	1.9686	6.4397	0.35	13.12
	Model	Fixed effects		0.05871	4.1792	4.2292		
		Random effects			−5.3181	13.7264		

*R*_*z*_	As-sintered		2.2383	0.02714	2.2098	2.2668	2.20	2.28
	SIE		2.3117	0.03710	2.2727	2.3506	2.26	2.36
	LPPA		12.2333	0.06154	12.1688	12.2979	12.15	12.31
	FS		57.5183	0.27701	57.2276	57.8090	56.98	57.74
	Total		18.5754	23.34012	8.7197	28.4311	2.20	57.74
	Model	Fixed effects		0.14373	18.5142	18.6366		
		Random effects			−23.4058	60.5566		

Pore area	As-sintered		8.6033	0.01211	8.5906	8.6160	8.59	8.62
	SIE		6.8633	0.01033	6.8525	6.8742	6.85	6.88
	LPPA		6.1500	0.00894	6.1406	6.1594	6.14	6.16
	FS		9.1883	0.01169	9.1761	9.2006	9.17	9.20
	Total		7.7013	1.26498	7.1671	8.2354	6.14	9.20
	Model	Fixed effects		0.01084	7.6966	7.7059		
		Random effects			5.4260	9.9765		

Pore diameter	As-sintered		0.0133	0.00516	0.0079	0.0188	0.01	0.02
	SIE		0.0100	0.00000	0.0100	0.0100	0.01	0.01
	LPPA		0.0167	0.00516	0.0112	0.0221	0.01	0.02
	FS		0.0133	0.00516	0.0079	0.0188	0.01	0.02
	Total		0.0133	0.00482	0.0113	0.0154	0.01	0.02
	Model	Fixed effects		0.00447	0.0114	0.0152		
		Random effects			0.0090	0.0177		

Bulk density	As-sintered		5.2400	0.00000	5.2400	5.2400	5.24	5.24
	SIE		5.1200	0.00632	5.1134	5.1266	5.11	5.13
	LPPA		6.0000	0.00000	6.0000	6.0000	6.00	6.00
	FS		6.1400	0.00000	6.1400	6.1400	6.14	6.14
	Total		5.6250	0.45943	5.4310	5.8190	5.11	6.14
	Model	Fixed effects		0.00316	5.6237	5.6263		
		Random effects			4.7986	6.4514		

Porosity	As-sintered		16.0600	0.09695	15.9583	16.1617	15.92	16.19
	SIE		10.8583	0.07627	10.7783	10.9384	10.75	10.95
	LPPA		13.5017	0.26992	13.2184	13.7849	12.98	13.75
	FS		20.0017	0.15145	19.8427	20.1606	19.75	20.17
	Total		15.1054	3.44851	13.6492	16.5616	10.75	20.17
	Model	Fixed effects		0.16659	15.0345	15.1764		
		Random effects			8.9089	21.3019		

VHN	As-sintered		920.0000	7.07107	912.5794	927.4206	910.00	930.00
	SIE		1346.0000	11.26055	1334.1828	1357.8172	1330.00	1360.00
	LPPA		1608.0000	28.64263	1577.9414	1638.0586	1576.00	1651.00
	FS		2137.0000	68.01470	2065.6229	2208.3771	2055.00	2220.00
	Total		1502.7500	451.72272	1312.0042	1693.4958	910.00	2220.00
	Model	Fixed effects		37.49400	1486.7852	1518.7148		
		Random effects			692.6738	2312.8262		

## References

[1] Miyazaki T, Nakamura T, Matsumura H, Ban S, Kobayashi T (2013). Current status of zirconia restoration. *Journal of Prosthodontic Research*.

[2] Piconi C, Maccauro G (1999). Zirconia as a ceramic biomaterial. *Biomaterials*.

[3] Garvie RC, Hannink RH, Pascoe RT (1975). Ceramic steel?. *Nature*.

[4] Piconi C, Burger W, Richter HG (1998). Y-TZP ceramics for artificial joint replacements. *Biomaterials*.

[5] Christel P, Meunier A, Heller M, Torre JP, Peille CN (1989). Mechanical properties and short-term in-vivo evaluation of yttrium-oxide-partially-stabilized zirconia. *Journal of Biomedical Materials Research*.

[6] Stadlinger B, Hennig M, Eckelt U, Kuhlisch E, Mai R (2010). Comparison of zirconia and titanium implants after a short healing period. A pilot study in minipigs. *International Journal of Oral and Maxillofacial Surgery*.

[7] Cochran DL (1999). A comparison of endosseous dental implant surfaces. *Journal of Periodontology*.

[8] Sennerby L, Roos J (1998). Surgical determinants of clinical success of osseointegrated oral implants: a review of the literature. *International Journal of Prosthodontics*.

[9] Chevalier J (2006). What future for zirconia as a biomaterial?. *Biomaterials*.

[10] Hisbergues M, Vendeville S, Vendeville P (2009). Review zirconia: Established facts and perspectives for a biomaterial in dental implantology. *Journal of Biomedical Materials Research. Part B Applied Biomaterials*.

[11] Ferguson SJ, Langhoff JD, Voelter K (2008). Biomechanical comparison of different surface modifications for dental implants. *International Journal of Oral and Maxillofacial Implants*.

[12] Aparicio C, Gil FJ, Fonseca C, Barbosa M, Planell JA (2003). Corrosion behaviour of commercially pure titanium shot blasted with different materials and sizes of shot particles for dental implant applications. *Biomaterials*.

[13] Aboushelib MN, Kleverlaan CJ, Feilzer AJ (2007). Selective infiltration-etching technique for a strong and durable bond of resin cements to zirconia-based materials. *Journal of Prosthetic Dentistry*.

[14] Salem NA, Abo Taleb AL, Aboushelib MN (2013). Biomechanical and histomorphometric evaluation of osseointegration of fusion-sputtered zirconia implants. *Journal of Prosthodontics*.

[15] Mendonça G, Mendonça DBS, Aragão FJL, Cooper LF (2008). Advancing dental implant surface technology—from micron- to nanotopography. *Biomaterials*.

[16] Biggs MJ, Richards RG, Dalby MJ (2010). Nanotopographical modification: a regulator of cellular function through focal adhesions. *Nanomedicine*.

[17] Vagkopoulou T, Koutayas SO, Koidis P, Strub JR (2009). Zirconia in dentistry: part 1. Discovering the nature of an upcoming bioceramic. *The European Journal of Esthetic Dentistry*.

[18] Serkan S, Onjen T, Gamze A (2013). Basic properties and types of zirconia: an overview. *World Journal of Stomatology*.

[19] Lange FF (1989). Powder processing science and technology for increased reliability. *Journal of the American Ceramic Society*.

[20] Rashad M, Gihan A, Elshenawy H (2013). Effect of surface treatment of copy milled zirconia ceramic restorations on bonding to resin cement. *Journal of Applied Sciences Research*.

[21] Re D, Augusti D, Augusti G, Giovannetti A (2012). Early bond strength to low-pressure sandblasted zirconia: evaluation of a self-adhesive cement. *The European Journal of Esthetic Dentistry*.

[22] Wolfart M, Lehmann F, Wolfart S, Kern M (2007). Durability of the resin bond strength to zirconia ceramic after using different surface conditioning methods. *Dental Materials*.

[23] Phark JH, Duarte S, Hernandez A, Blatz MB, Sadan A (2009). In vitro shear bond strength of dual-curing resin cements to two different high-strength ceramic materials with different surface texture. *Acta Odontologica Scandinavica*.

[24] Queiroz JRC, Paulo GP, Özcan M, Nogueira L (2012). Effect of airborne particle abrasion protocols on surface topography of Y-TZP ceramic. *Cerâmica*.

[25] Tanaka R, Fujishima A, Shibata Y, Manabe A, Miyazaki T (2008). Cooperation of phosphate monomer and silica modification on zirconia. *Journal of Dental Research*.

[26] Kern M, Barloi A, Yang B (2009). Surface conditioning influences zirconia ceramic bonding. *Journal of Dental Research*.

[27] Sun R, Suansuwan N, Kilpatrick N, Swain M (2000). Characterisation of tribochemically assisted bonding of composite resin to porcelain and metal. *Journal of Dentistry*.

[28] Matějíček J, Kolman B, Dubský J, Neufuss K, Hopkins N, Zwick J (2006). Alternative methods for determination of composition and porosity in abradable materials. *Materials Characterization*.

[29] He Y, Winnubst L, Burggraaf AJ, Verweij H, van der Varst PGT, de With B (1997). Influence of porosity on friction and wear of tetragonal zirconia polycrystal. *Journal of the American Ceramic Society*.

[30] Packham DE (2003). Surface energy, surface topography and adhesion. *International Journal of Adhesion and Adhesives*.

[31] Pittayachawan P, McDonald A, Petrie A, Knowles JC (2007). The biaxial flexural strength and fatigue property of Lava Y-TZP dental ceramic. *Dental Materials*.

[32] Özcan M, Nijhuis H, Valandro LF (2008). Effect of various surface conditioning methods on the adhesion of dual-cure resin cement with MDP functional monomer to zirconia after thermal aging. *Dental Materials Journal*.

[33] Kosmač T, Oblak C, Jevnikar P, Funduk N, Marion L (1999). The effect of surface grinding and sandblasting on flexural strength and reliability of Y-TZP zirconia ceramic. *Dental Materials*.

